# Brain Metal Distribution and Neuro-Inflammatory Profiles after Chronic Vanadium Administration and Withdrawal in Mice

**DOI:** 10.3389/fnana.2017.00058

**Published:** 2017-07-25

**Authors:** Oluwabusayo R. Folarin, Amanda M. Snyder, Douglas G. Peters, Funmilayo Olopade, James R. Connor, James O. Olopade

**Affiliations:** ^1^Department of Medical Laboratory Science, Ladoke Akintola University of Technology Osogbo, Nigeria; ^2^Department of Neurosurgery, Pennsylvania State College of Medicine Hershey, PA, United States; ^3^Department of Anatomy, University of Ibadan Ibadan, Nigeria; ^4^Department of Veterinary Anatomy, University of Ibadan Ibadan, Nigeria

**Keywords:** vanadium, LA–ICP–MS, neuro-inflammation, neurotoxicity, withdrawal

## Abstract

Vanadium is a potentially toxic environmental pollutant and induces oxidative damage in biological systems including the central nervous system (CNS). Its deposition in brain tissue may be involved in the pathogenesis of certain neurological disorders which after prolonged exposure can culminate into more severe pathology. Most studies on vanadium neurotoxicity have been done after acute exposure but in reality some populations are exposed for a lifetime. This work was designed to ascertain neurodegenerative consequences of chronic vanadium administration and to investigate the progressive changes in the brain after withdrawal from vanadium treatment. A total of 85 male BALB/c mice were used for the experiment and divided into three major groups of vanadium treated (intraperitoneally (i.p.) injected with 3 mg/kg body weight of sodium metavanadate and sacrificed every 3 months till 18 months); matched controls; and animals that were exposed to vanadium for 3 months and thereafter the metal was withdrawn. Brain tissues were obtained after animal sacrifice. Sagittal cut sections of paraffin embedded tissue (5 μm) were analyzed by the Laser ablation-inductively coupled plasma-mass spectrometry (LA–ICP–MS) to show the absorption and distribution of vanadium metal. Also, Haematoxylin and Eosin (H&E) staining of brain sections, and immunohistochemistry for Microglia (Iba-1), Astrocytes (GFAP), Neurons (Neu-N) and Neu-N + 4′,6-diamidine-2′-pheynylindole dihydrochloride (Dapi) Immunofluorescent labeling were observed for morphological and morphometric parameters. The LA–ICP–MS results showed progressive increase in vanadium uptake with time in different brain regions with prediction for regions like the olfactory bulb, brain stem and cerebellum. The withdrawal brains still show presence of vanadium metal in the brain slightly more than the controls. There were morphological alterations (of the layering profile, nuclear shrinkage) in the prefrontal cortex, cellular degeneration (loss of dendritic arborization) and cell death in the Hippocampal CA1 pyramidal cells and Purkinje cells of the cerebellum, including astrocytic and microglial activation in vanadium exposed brains which were all attenuated in the withdrawal group. With exposure into old age, the evident neuropathology was microgliosis, while progressive astrogliosis became more attenuated. We have shown that chronic administration of vanadium over a lifetime in mice resulted in metal accumulation which showed regional variabilities with time. The metal profile and pathological effects were not completely eliminated from the brain even after a long time withdrawal from vanadium metal.

## Introduction

Vanadium (V) is a metalloid widely distributed in the environment and it exerts potent toxic effects on a wide variety of biological systems. While some derivatives of vanadium have been found to be useful in medicine and industry (Ray et al., 2006), environmental and occupational exposure to this metal continues to be a health risk to humans and animals (Shrivastava et al., 2007).

Exposure to neurotoxic metals such as vanadium occurs through various sources including heavy metals mining (Moskalyk and Alfantazi, 2003), combustion products of vanadium bearing fuel oils (Amorim et al., 2007), forest fires and volcanic emissions; in addition, large quantities of vanadium compounds have been reported to be released into the environment mainly through the burning of fossil fuels having vanadium contaminated crude as seen in oil producing communities such as Venezuela, the Arabian Gulf, the Gulf of Mexico and the Nigerian Niger Delta (Olopade and Connor, 2011; Saxena et al., 2013; Fortoul et al., 2014). In addition, vanadium accumulates in the soil, groundwater and plants that may be consumed by both animals and humans (Pyrzyńska and Wierzbicki, 2004).

Current opinion is that vanadium induces oxidative stress caused by reactive oxygen species (ROS) generation *in vitro*, as well as lipid peroxidation and oxidative damage and this action has been strongly linked to vanadate induced effect in biological systems (Evangelou, 2002; García et al., 2004), and this in turn may cause neurotoxicity in humans and animals (Haider et al., 1998; Chen et al., 2001; García et al., 2004; Olopade et al., 2011). Earlier studies have shown that vanadium crosses the blood brain barrier (García et al., 2004) to induce neuropathology including neurobehavioral (Li et al., 2013; Saxena et al., 2013; Mustapha et al., 2014; Folarin et al., 2016), neurochemical (Sasi et al., 1994; García et al., 2004) and neurocellular (Domingo, 1996; Garcia et al., 2005; Avila-Costa et al., 2006) changes. In humans, features of acute neurotoxicity include central nervous system (CNS) perturbations, (CNS) depression, tremor, impaired conditioned reflexes, as well as congestion of brain and spinal cord (Haider et al., 1998; Soazo and Garcia, 2007).

Most animal experiments on neuro-cellular studies involving vanadium metal have been based on acute exposure while in reality many people occupationally (Fortoul et al., 2014) and environmentally (Olopade and Connor, 2011) exposed to vanadium are so exposed for decades or even a life time. Few studies have reported progressive neuro-cellular and neuro-inflammatory changes induced by long term vanadium exposure. Azeez et al. (2016) showed functional deficit, glial cell activation and region-dependent myelin damage in the brain of mice after 90 days of postnatal vanadium exposure. Our previous work has shown that a life time administration of vanadium in mice leads to memory deficits and progressive recovery after long period of treatment withdrawal (Folarin et al., 2016).The present study is designed to assess brain vanadium distribution patterns, and cellular (glial and neuronal) injury after a long term exposure, as well as the related progressive changes in the brain after withdrawal from treatment.

## Materials and Methods

### Animal Experiments

All experiments were approved and carried out in accordance to the guidelines of the animal use and ethics committee of the University of Ibadan, ethical code number UI-ACUREC/App/2016/011.

### Experimental Design

A total of 85 male BALB/c mice (4 weeks old) were used for the experiment which covered a period of 18 months. The animals were bred and housed in the experimental animal house of the Neuroscience unit of the Department of Veterinary Anatomy, University of Ibadan. The animals were pellet-fed, given tap water *ad libitum* and were kept at 27°C with natural light and dark cycles. The animals were assigned to one of the following animal groups: vanadium- (V-) treated, control and withdrawal groups.

#### Animal Design

V-treated group consisted of six subgroups of 12 animals. The subgroups are designated as V3, V6, V9, V12, V15 and V18. The mice (from 4 weeks of age) were intraperitoneally (i.p.) administered with 3 mg/kg b.w/day of vanadium (sodium metavanadate, Sigma-Aldrich, St. Louis, MO, USA), i.p. thrice a week for 3, 6, 9, 12, 15 and 18 months. This dose and route of administration is based on the findings of García et al. (2004) as it is neurotoxic with minimal mortalities. Sample mice were sacrificed every 3 months until the animals were 18 months post exposure.

Control group consisted of six subgroups of 12 animals. The subgroups are designated as C3, C6, C9, C12, C15 and C18. The mice (from 4 weeks of age) were intraperitoneally administered with sterile water, i.p. thrice a week for 3, 6, 9, 12, 15 and 18 months which was volume matched with the V-treated group. Sample mice were sacrificed as above.

Withdrawal group consisted of five subgroups of 12 animals. The subgroups are designated as W3, W6, W9, W12 and W15. The mice (from 4 weeks old) were intraperitoneally administered with 3 mg/kg b.w./day of vanadium (sodium metavanadate Sigma-Aldrich, St. Louis, MO, USA), i.p. thrice a week only for the first 3 months and then vanadium administration was stopped. Subsequently, the animals were treated as done in controls. Sample mice were sacrificed after withdrawal from treatment every 3 months till 18 months.

#### Sample Collection

The mice were anesthetized with ketamine and then perfused transcardially with 4% phosphate buffered formalin with the aid of a perfusion pressure pump and brains were removed according to the method described by Olopade et al. (2011). Briefly, the frontal, parietal and temporal bones were removed to expose the brain which was gently scooped out, post-fixed for 4 h in the same solution, then processed and embedded in paraffin blocks as described by Mustapha et al. (2014). Sections were cut on a standard microtome at 5-μm thickness from paraffin embedded tissue, sectioned at Sagittal level 17, (Bregma lateral coordinates 0.675 μm, from Allen reference Mouse Brain Atlas, 2016), containing the neocortex, basal ganglia, midbrain and brain-stem.

#### Sample Preparation for LA–ICP–MS

Cut sections were mounted on silane-coated soda-glass microscope slides (StarFrost^®^; ProSciTech, USA). Sections were dewaxed in xylene (Sigma, USA) and decreasing concentrations of ethanol (Sigma, USA) in water according to standard protocols. Samples were finally washed in deionized water (18.2 MΩ; Merk Millipore) and dried at room temperature before analysis.

#### LA–ICP–MS Imaging

The LA–ICP–MS was done according to the method described by Hare et al. (2009, 2012). Briefly, sections imaged at 80 μm spatial resolution (total area = 6.4 mm^2^/pixel) were ablated with a 193 nm New Wave excimer ArF source laser, which was fitted with a “TwoVol2” ablation cell with collection cup just above the ablation spot. The laser unit was connected to a Thermo X-Series II quadrupole ICP–MS instrument. The instrument contained Xt cones at the plasma interface. The sample cone was Ni with a Cu core and the skimmer cone was Ni. The collision cell was turned off to increase sensitivity. We tuned the quadrupole while ablating NIST 612 glass standard, attempting to maximize signal while keeping 238U/232Th ratio near 1.2 and minimizing 248ThO/232Th below 3%. We set a low energy fluence of 0.4 J/cm^2^ and laser power of 3% combined with a high repetition rate of 20 Hz to ensure proper ablation of tissue. Ablation lines were set parallel without any overlap. We ablated the tissue at 320 μm/s and measured ^13^C, ^31^P, ^51^V, ^52^Cr and ^53^ClO with a sampling rate of 0.25 s so that each voxel contained a single sampling of each element. Qualitative counts for V were compared to Cr to ClO to ensure there wasn’t a false positive signal, and age matched control tissue was ablated at the same time to validate any observable difference in the experimental group.

#### Immunohistochemistry

Paraffin sections were dewaxed, rehydrated and immersed in distilled water. Antigen retrieval was done in 10 mM citrate buffer (pH = 6.0) for 25 min, with subsequent peroxidase quenching in 3% H_2_O_2_/methanol. All the sections were blocked in 2% milk for 1 h and probed with the following antibodies overnight: anti-GFAP Rabbit Polyclonal antibody for astrocytic morphology (1:1000; Dako, Denmark), anti-NeuN Rabbit monoclonal antibody for neuronal morphology (1:3000; Abcam, Cambridge, MA, USA) and anti-Iba-1 Rabbit polyclonal antibody for microglia morphology (1:1000; Abcam, Cambridge, MA, USA) for 16 h at 4°C. After washing, the sections were incubated for 2 h at room temperature in the appropriate biotinylated secondary antibodies (diluted 1:200; all purchased from Vector Labs). The sections were then reacted in avidin-biotin-peroxidase solution (ABC kit, Vectastain, Vector Labs, USA) using 3,30-diaminobenzidine as chromogen, according to manufacturer’s protocol. Images were acquired with Nikon bright-field microscope equipped with digital camera. For NeuN immuno-fluorescent labeling, sections were probed with anti-NeuN mouse monoclonal antibody (1:1000; Abcam, Cambridge, MA, USA) diluted at optimal working concentrations in 1.5% Normal Goat Serum (NGS) in PBS for 16 h at 4°C, Sections were then incubated for 2 h with fluorescent-conjugated secondary antibodies (anti-mouse IgG Alexa Flour555 antibodies (Molecular Probes) at 1:150 dilution, 4′,6-diamidine-2′-pheynylindole dihydrochloride, (DAPI; 1:1000; Invitrogen, USA) staining was used in all fluorescence staining conditions to identify nuclear DNA in the cell type.

#### Image Analysis

To avoid experimenter bias during all phases of image collection and analysis, slides were coded and experimenter was blind to animal condition. Prefrontal cortex, dorsal CA1 and CA3 region, genu of corpus callosum and white matter of the cerebellum was imaged using a spinning disc laser confocal system (Nikon Eclipse 80i.) equipped with ×4 ×10 and ×40 dry and ×100 oil objectives connected to a camera (Nikon DS-Fi1, NIS-Elements BR 3.2 software). Identical light intensity and exposure settings were applied to all images taken for each experimental set. The brain regions studied were chosen because of previous studies which showed vanadium leads to memory loss (Folarin et al., 2016) and loss of motor function (García et al., 2004). The prefrontal cortex and hippocampal region are cognition centers and are associated with chronic metal pollution and neurodegenerative changes (Calderón-Garcidueñas et al., 2012).

#### Stereological Analysis

Immunostaining was quantified using standardized stereological method based on “systematic random sampling”. Astrocytes and microglia lying within the CA1 region of the hippocampus, genu of corpus callosum and white matter of the cerebellum were counted manually by two blinded investigators using ×20 images, and compared blindly between control, vanadium treated and the withdrawal groups. For each mouse Neu-N immunolabeling was quantitatively analyzed using ×20 and ×40 images within the prefrontal and hippocampal region of CA1 and CA3 to show pyknosis and neuronal loss, the number of undegenerated purkinje cells in the cerebellar cortex from H and E stained slides were also counted and analyzed using ImageJ analysis.

#### Statistical Analysis

All data were expressed as mean ± standard deviation. Comparison between groups was performed using one way analysis of variance (ANOVA). A *p* < 0.05 was considered significant. All statistical analyses were carried out using GraphPad Prism Version 4 (GraphPad Software, San Diego, CA, USA).

## Results

**Figure 1:**
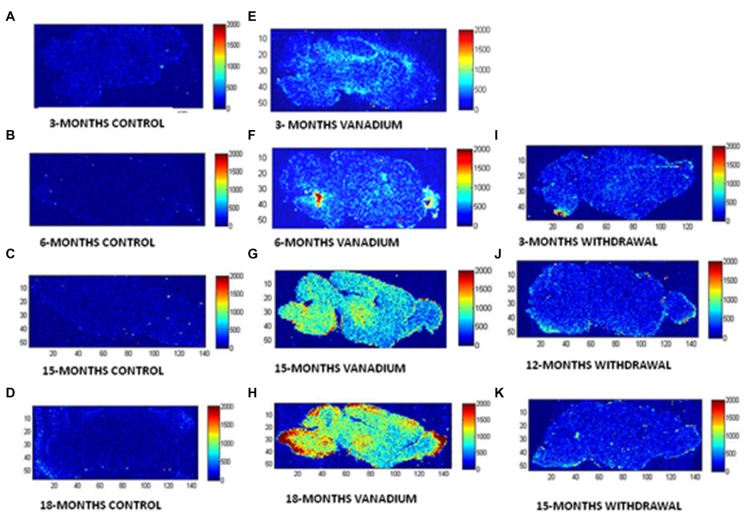
Laser ablation-inductively coupled plasma-mass spectrometry (LA–ICP–MS) is used to reveal and quantify metals in brain tissue. To show the microspatial distribution of vanadium metal within anatomical regions of mice brain after chronic exposure. BASAL INDICES: 80 μm × 80 μm square laser spot, 3% laser power with 0.4 J/cm^2^ confluence, 320 μm/s laser speed, 20 Hz, 0.25 s Integration. (**A–D**, control brains), (**E–H**, vanadium exposed brains), (**I–K**, withdrawal brains) at 3, 6, 15 and 18 months time points of treatments.

The LA–ICP–MS showed evidence that vanadium crosses the Blood Brain Barrier, enters the brain tissue and is deposited in different regions (see Figure 1). The LA–ICP–MS results also showed progressive increase in vanadium uptake with time in the treated brains relative to the controls while the withdrawal group showed marked elimination of vanadium metal from the brain indicated by reduced relative intensities in comparison with the treated groups.

**Figure 2:**
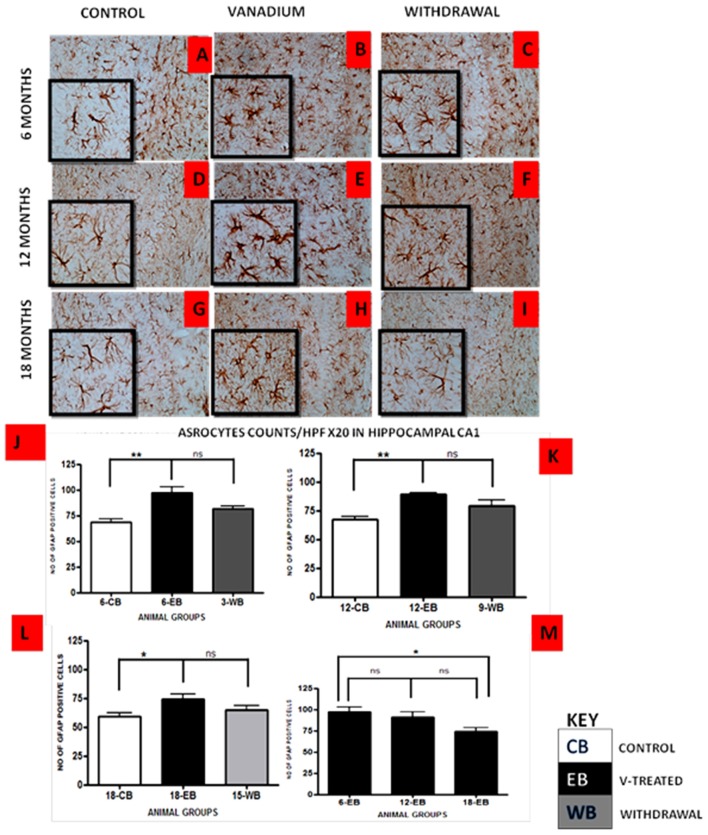
GFAP-immunostained dorsal CA1 hippocampal region of vanadium exposed **(B,E,H)**, age matched control **(A,D,G)** and withdrawal group **(C,F,I)** mice. Vanadium treatments for 6, 12 and 18 months revealed Astrocytic activation identified by thickened cell body with more extended cytoplasmic processes relative to the matched controls while the withdrawal groups showed reversal effect relative to vanadium exposed groups. The number of GFAP positive cells were counted by two blinded investigators and average of *N* = 5 per group counts are shown per high powered field (HPF, 20×) in **(D–F)** respectively. The differences between groups **(J–L)** were evaluated for significance using analysis of variance (ANOVA; *p < 0.05; **p < 0.01). Astrocytic response decreases into old age with increasing vanadium exposure (**M**; 6EB vs. 18EB = *p < 0.05). Magnification: ×200, inset: ×400.

**Figure 3:**
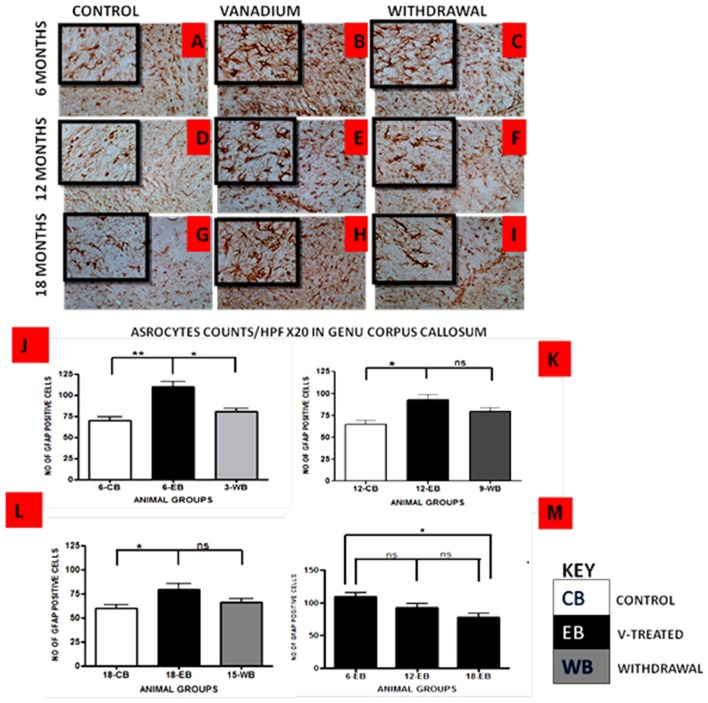
GFAP-immunostained Genus corpus callosum of vanadium exposed **(B,E,H)**, age matched control **(A,D,G)** and withdrawal group **(C,F,I)** mice. Vanadium treatments for 6, 12 and 18 months revealed astrocytic activation identified by thickened cell body with more tortuous cytoplasmic processes relative to the matched controls while the withdrawal groups showed reversal effect relative to vanadium exposed groups. The number of GFAP positive cells were counted by two blinded investigators and average of *N* = 5 per group counts are shown per HPF (20×) in **(D–F)** respectively. The differences between groups **(J–L)** were evaluated for significance using ANOVA (*p < 0.05; **p < 0.01). Astrocytic response decreases into old age with increasing vanadium exposure (**M**; 6EB vs. 18EB = *p < 0.05). Magnification: ×200, inset: ×400.

**Figure 4:**
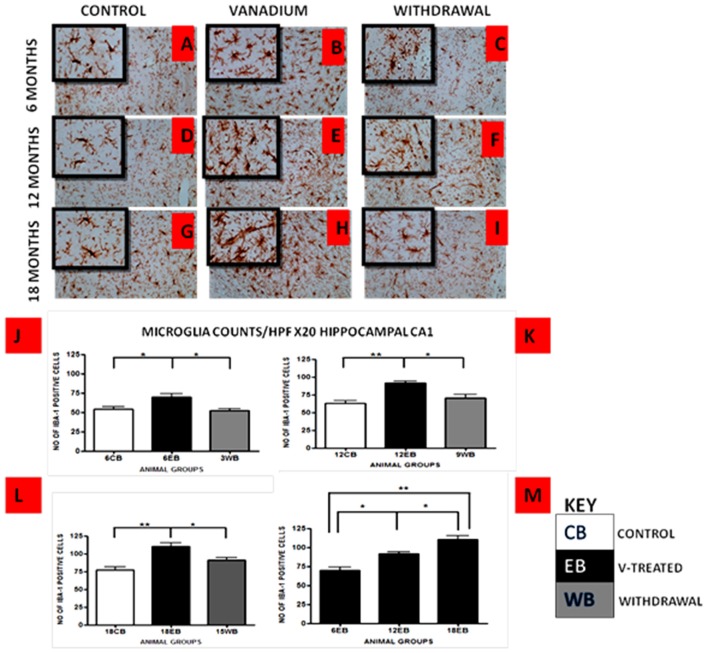
IBA-1 immunostained dorsal CA1 hippocampal region of vanadium exposed **(B,E,H)**, age matched control **(A,D,G)** and withdrawal group **(C,F,I)** mice after intermittent vanadium treatments for 6, 12 and 18 months revealed microglial activation identified by an enlarged cell body with several short, thickened processes, relative to the matched controls with longer, finer branches while the withdrawal groups showed better morphology relative to vanadium exposed groups. Figure 5 **(J–L)**, the number of IBA-1 positive cells in hippocampal CA1 region, were significantly increased in all the vanadium exposed groups compared to control, while the withdrawal groups showed reversal effect. Microglia activation increases into advanced age with increasing vanadium exposure (**M**; 6EB vs. 18EB = **p < 0.01), (ANOVA: *p < 0.05, **p < 0.01). Magnification: ×200 inset: ×400.

**Figure 5:**
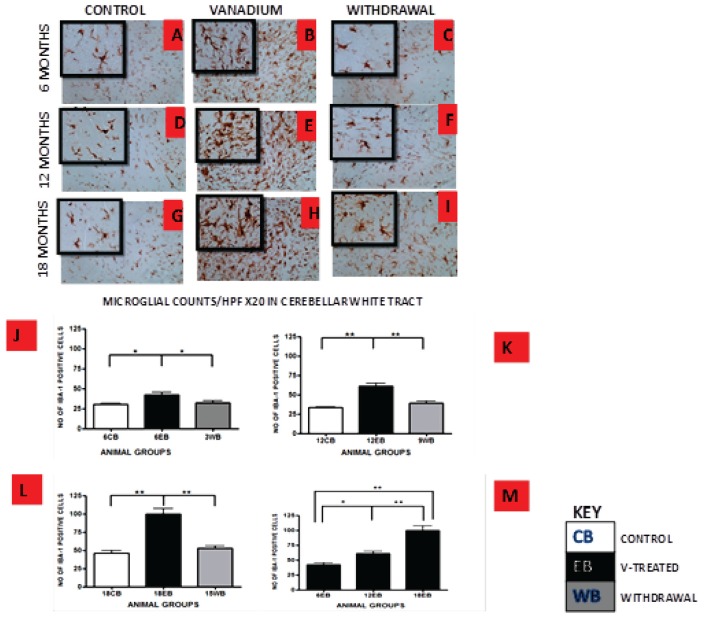
IBA-1 immuno-stained white matter of cerebellum of vanadium exposed **(B,E,H)**, age matched control **(A,D,G)** and withdrawal group **(C,F,I)** mice after intermittent vanadium treatments for 6, 12 and 18 months revealed microglial activation identified by an enlarged cell body with several short, thickened processes, relative to the matched controls with longer, finer branches while the withdrawal groups showed better morphology relative to vanadium exposed groups. **(J–L)** The number of IBA-1 positive cells in hippocampal CA1 region, and cerebellum were significantly increased in all the vanadium exposed groups compared to controls, while the withdrawal groups showed reversal effect. Persistent microglia activation throughout the exposure increases to phagocytic state indicated by amoeboid isoform. **(B,E,H)** Microglia activation increases into advanced age with increasing vanadium exposure (**M**; 6EB vs. 18EB = **p < 0.01), (ANOVA: *P < 0.05, **p < 0.01). Magnification: ×200 inset: ×400.

Vanadium treatments for 3–18 months resulted in astrocytic activation in the dorsal hippocampal CA1 region and genu of corpus callosum. GFAP immunoreactive cells displayed thickened cell body with more extensive cytoplasmic processes in the vanadium exposed groups (Figures 2, 3B,E,H) relative to the age matched controls (Figures 2, 3A,D,G) while the withdrawal groups (Figures 2, 3C,F,I) showed less reactivity relative to vanadium exposed groups. Quantitative analysis of GFAP immunostaining also confirmed these observations (Figures 3J–L), however in the withdrawal groups, the analysis revealed non-significant decrease in astrocytic number relative to the vanadium treated groups. This result also showed that astrocytic response decreases with increasing age and period of exposure (Figures 2, 3M). IBA-1 (microglia) immunoreactive cells in the vanadium exposed groups (Figures 4, 5B,E,H) were markedly larger with several short, thickened processes, relative to the control brains (Figures 4, 5A,D,G) with microglial cells in resting state, while the withdrawal brains (Figures 4, 5C,F,I) showed less reactivity relative to the vanadium treated brains. Microglial activation occurred progressively and reaches phagocytic state indicated by amoeboid isoform (Figure 5M). Microglial cell count/HPF of hippocampal CA1 region and white matter of the cerebellum (Figures 4, 5J–L) were significantly elevated in vanadium exposed brains relative to the controls while the withdrawal groups showed less reactivity compared to the treated groups. This result also showed that microgliosis increased with increasing age and period of exposure (Figures 4, 5M).

**Figure 6:**
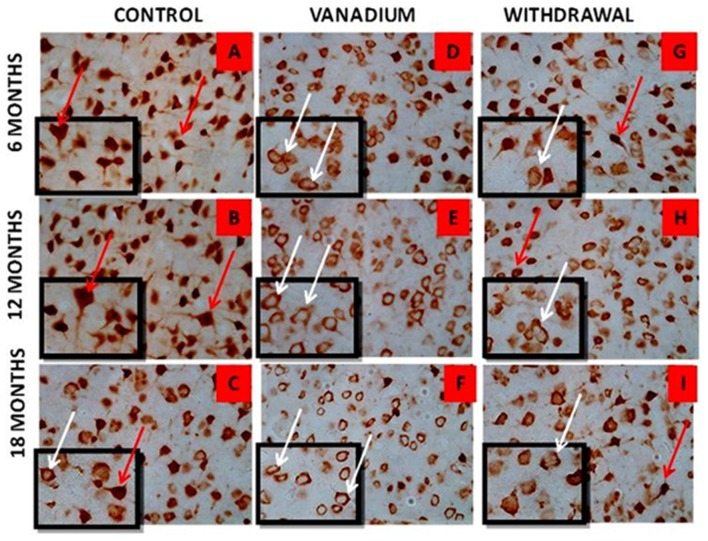
NeuN immuno-histochemistry revealed the cytotoxic effect of vanadium on the pyramidal cells of the prefrontal cortex after chronic exposure. The cortical pyramidal cells showed morphological alterations including pyknosis, cell clustering, loss of layering pattern and cytoplasmic vacuolation in the vanadium exposed groups (white arrows in **B,E,H**) relative to the control (red arrows in **A,D**) with normal neuronal morphology. The withdrawal groups **(C,F,I)** showed reversal effect with less cellular toxicity relative to vanadium exposed groups. The vanadium cytotoxicity was also observed in the aged control mice brain (white arrows in **G**), indicative of neuronal degeneration seen also at early vanadium exposure. Magnification: ×400, inset: ×600.

**Figure 7:**
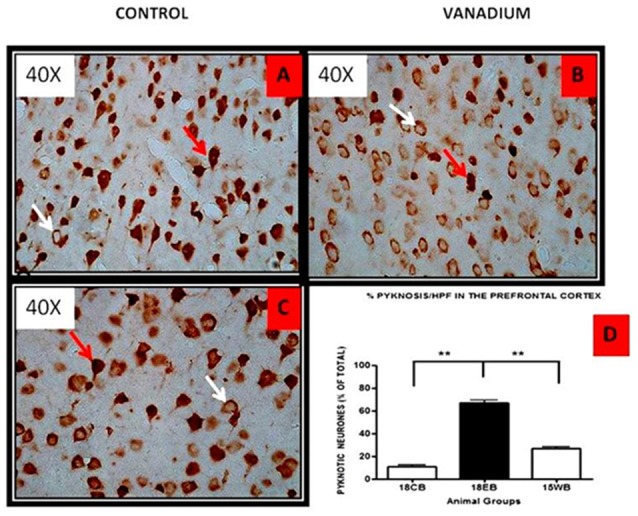
HPF photomicrographs showing the pyramidal cells of the prefrontal cortex using the NeuN immunohistochemistry. No remarkable abnormality was observed in the cortical sections from animals of the control groups. The normal neurons were identified by their rounded and pale nuclei (see **A** red arrow), whereas degenerating neurons had smaller cell bodies and pyknotic nuclei (see **B** white arrow). There was evidence of vacuolation of neuropil surrounding the degenerating neurons. The withdrawal brain **(C)** showed lesscellular pathology relative to the exposed brains. Quantitative analysis **(D)** showed that the mean % pyknosis of vanadium exposed groups were significantly (**P < 0.001) elevated relative to the control brain while the withdrawal groups were significantly (**P < 0.001) less than vanadium exposed groups. Magnification: ×400.

**Figure 8:**
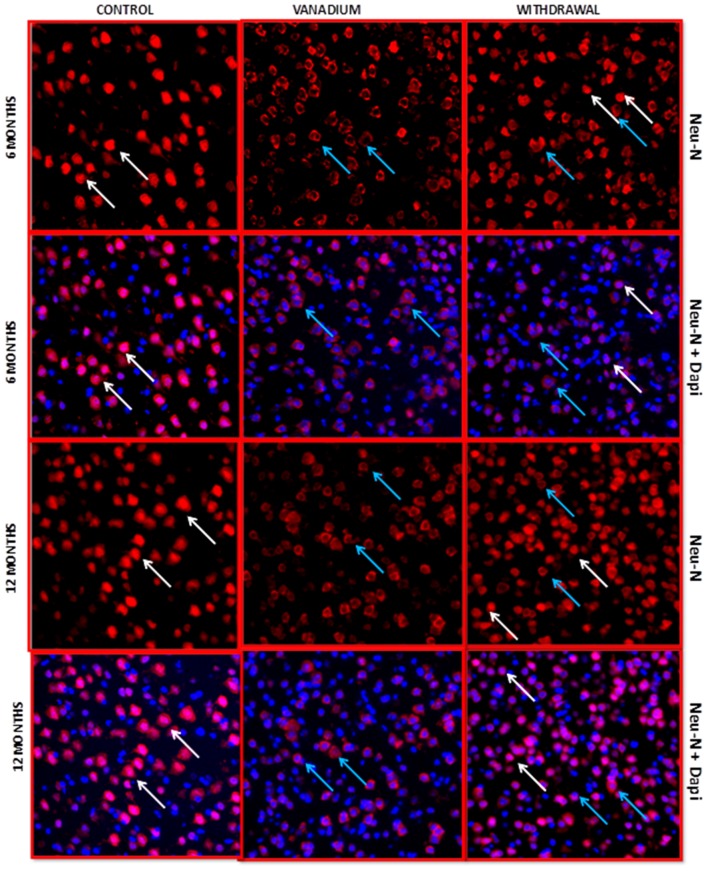
NeuN + 4′,6-diamidine-2′-pheynylindole dihydrochloride (DAPI) Immuno-fluorescent labeling of the prefrontal cortex revealed the fate of the nucleus after chronic vanadium exposure. NeuN colocalization with Dapi revealed pyramidal cells with intact but shrunken nucleus and cytoplasmic loss (blue arrows) relative to the control groups with normal morphology (white arrows), chronic vanadium exposure resulted in cytotoxicity which appear more pronounced than its apoptotic effects. Sections were double-labeled with NeuN antibody in conjunction with DAPI (blue), a nuclear DNA cell marker, and imaged at ×20 magnifications using confocal microscopy. Magnification: ×200.

**Figure 9:**
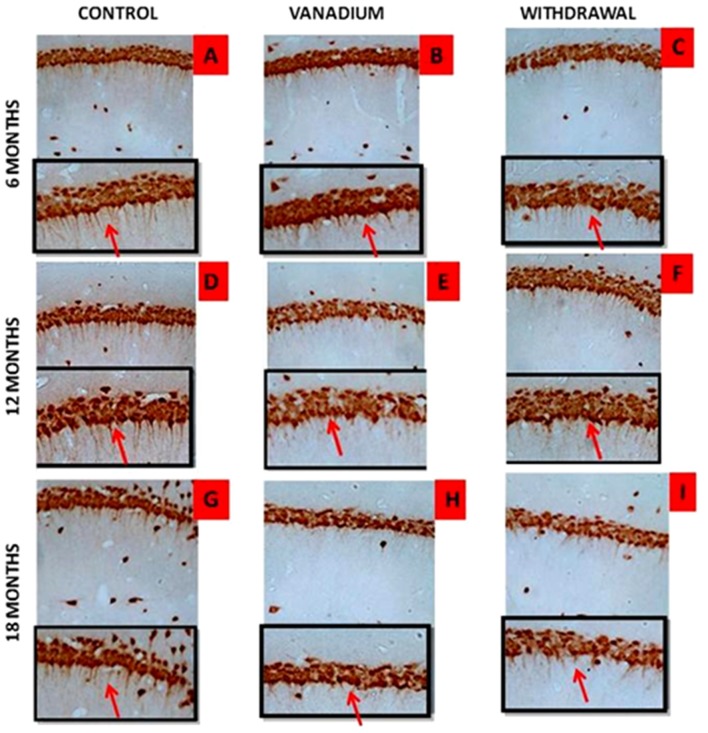
Dendritic arborization is lost with increasing vanadium exposure. NeuN immunohistochemistry revealed progressive loss of apical dendrites of the pyramidal cells of the dorsal hippocampal CA1 region. In **(A,D,G)** control pyramidal cells of hippocampus CA1 are identified, in which are notorious the presence of apical dendrites relative to **(B,E,H)** with evidence of the dendritic loss after chronic V treatment. Withdrawal groups **(C,F,I)** showed reversal effect relative to vanadium exposed groups **(B,E,H)**. Magnification: ×200, inset: ×400.

**Figure 10:**
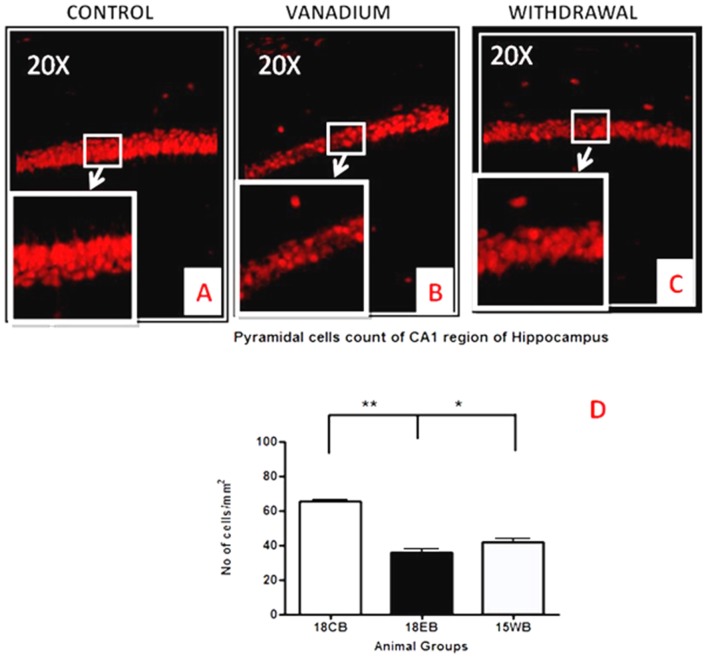
Pyramidal cell loss of the dorsal hippocampal CA1 region after chronic vanadium exposure. NeuN immuno-histochemistry showed evidence of neuronal loss after 18 months of vanadium treatments. Quantitative analysis **(D)** revealed significant (**P < 0.001) decrease in the no of pyramidal neurones in the exposed groups **(B)** relative to the control **(A)** while the withdrawal groups **(C)** showed reversal effect with significant (*P < 0.05) increase in neuronal no relative to vanadium exposed groups **(B)**. Magnification: ×200, inset: ×400.

**Figure 11:**
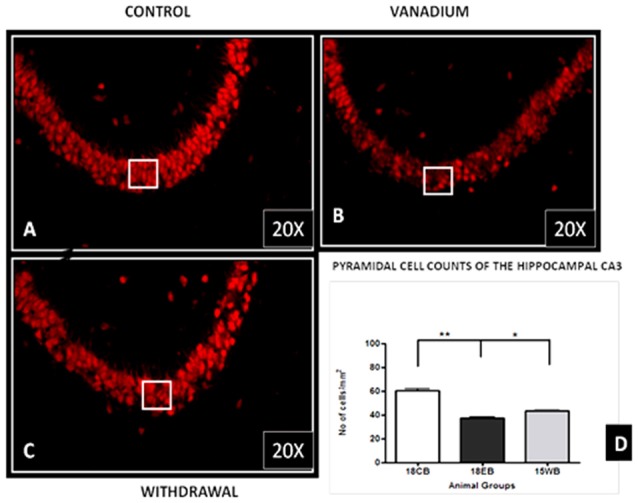
Pyramidal cell loss of the hippocampal CA3 region after chronic vanadium exposure. NeuN immuno-histochemistry showed evidence of neuronal loss after 18 months of exposure. Quantitative analysis **(D)** revealed significant (**P < 0.001) decrease in the number of pyramidal neurones in the exposed groups **(B)** relative to the control **(A)** while the withdrawal groups **(C)** showed reversal effect with significant (*P < 0.05) increase in neuronal no relative to vanadium exposed groups **(B)**. Magnification: ×200, inset: ×400.

**Figure 12:**
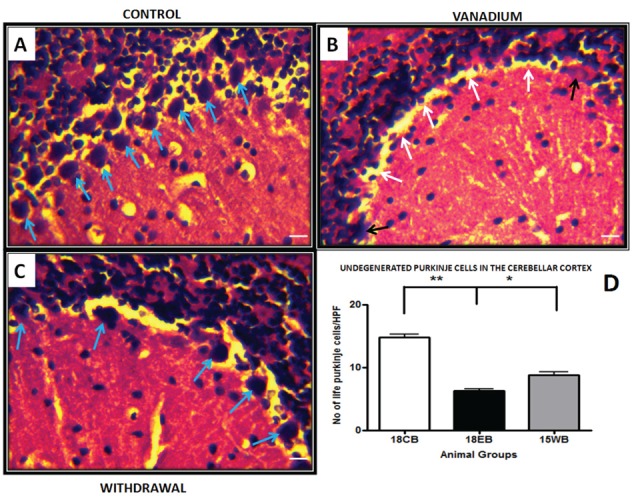
Haematoxylin and Eosin (H&E) stained sections of cerebellar cortex for control mice **(A)** and vanadium 18 months exposed mice **(B)** and age matched withdrawal **(C)**. **(A)** Shows numerous Purkinje cells (blue arrows) intact in the Purkinje cell layer. The white arrows in **(B)** shows Purkinje cells detachment and sloughed cells (black arrows) from Purkinje cell layer, the withdrawal groups **(C)** shows less cell detachment compares with V groups. Quantitative analysis **(D)** revealed significantly (**P < 0.001) reduced Purkinje cell count in the vanadium exposed group relative to the control, while the withdrawal group count was significantly (*P < 0.05) high relative to vanadium group **(D)**, scale bar 25 μm.

NeuN immunohistochemistry revealed damaged pyramidal cells of the prefrontal cortex with morphological alterations including pyknosis, cell clustering, loss of layering pattern and cytoplasmic vacuolation in the vanadium exposed brains (white arrows in Figures 6D–F) relative to the control (red arrows, Figures 6A,B) with normal neuronal morphology. The withdrawal groups (Figures 6G–I) showed reversal effect relative to vanadium exposed groups. The cellular pathology observed in the vanadium exposed brain were also seen in the aged control mice brain (white arrows in Figure 6C). Quantification of pyknotic neurons of the total NeuN positive cells in the prefrontal cortex of vanadium exposed groups was significantly elevated relative to the controls while the withdrawal groups was significantly less than vanadium exposed group (Figure 7). NeuN + Dapi immuno- fluorescent staining in prefrontal cortex was used to confirm the immunohistochemistry data. This co-localization revealed pyramidal cells with intact but shrunken nucleus and cytoplasmic loss (Figure 8). NeuN immunohistochemistry in hippocampal CA1 revealed progressive loss of apical dendrites of the pyramidal cells in vanadium exposed mice (Figures 9B,E,H) in comparison with controls (Figures 9A,D,G) while the withdrawal groups (Figures 9C,F,I) showed less dendritic loss relative to vanadium exposed groups. Quantification of the total NeuN immunoreactive cells in the CA1 and CA3 neurons showed that mean total cells of vanadium exposed groups was significantly reduced relative to the control brains while the withdrawal groups was significantly higher than vanadium exposed groups (Figures 10, 11). In addition, histology showed neuronal loss from the cerebellar cortex. The mean total count of undegenerated Purkinje cells of vanadium exposed groups was significantly reduced relative to the control brain (Figure 12) while the withdrawal groups was significantly higher than vanadium exposed groups.

## Discussion

The LA–ICP–MS showed evidence that vanadium crosses the Blood Brain Barrier, enters the brain parenchyma and is deposited in different regions. Cumulative evidence has revealed that the brain barriers are subject to toxic insults from heavy metal exposure (Zhen et al., 2002). Progressive increase in vanadium accumulation in the exposed brains is indicative of an increase in vanadium uptake into the brain over time. Avila-Costa et al. (2005) showed that vanadium accumulation in the brain after exposure depends more on the duration of exposure than the concentration of administration and also strongly correlated with the CNS damage induced. They further reported that the severity of ependymal epithelium disruption after vanadium pentoxide inhalation strongly correlated with the duration of exposure.

This to the best of our knowledge is the first report of LA–ICP–MS–based vanadium distribution in the brain of mice over a lifetime and has conclusively shown that vanadium metal crosses the Blood Brain Barrier and accumulates in the brain. Our study shows evidence that with chronic exposure, vanadium has a predilection for the olfactory bulb, brain stem and cerebellum. This supports the work of Haider et al. (1998), Garcia et al. (2005) and Ngwa et al. (2014) who reported specific pathologies in the olfactory bulb, brain stem and cerebellum respectively. Further work is ongoing by us to ascertain if predilection of accumulation translates to severity of pathologies relative to other brain regions.

This study has also shown evidence of clearance of vanadium metal from the brain after cessation of exposure indicating that following withdrawal, the brains have low potential for retention of the absorbed vanadium. While transferrin is known to actively transport vanadium into the brain (Usende et al., 2016), the exact mechanism of vanadium clearance from the brain needs to be investigated.

We investigated in this study microglia activation in response to chronic vanadium neurotoxicity in the hippocampus, genus of corpus callosum and cerebellum. The degree of microglial activation correlates with the duration of vanadium exposure and neuronal damage both in the hippocampal region and the cerebellum. This is indicative of the recruitment and activation of microglial cells by vanadium induced oxidative stress (Block and Calderón-Garcidueñas, 2009) resulting from free radicals production (Tsuda et al., 2004). The microglial activation persisted throughout the duration of exposure and reached a phagocytic state indicated by amoeboid isoforms mainly in the cerebellar white matter. Microglia activation has been reported to be readily triggered by environmental toxicant (Block and Hong, 2007) and may undergo a morphological change into amoeboid shape with short or non-existent processes (Kreutzberg, 1996) to favor phagocytosis and mobility. Upregulation of glial cells has also been associated with pathogenesis of senile neurodegenerative conditions like Alzheimer’s disease. Hence, we presume that in the present study, upregulation of glial cells could be suggestive of neurodegenerative effects in old age which was prematurely induced by vanadium metal.

Astrocytes play a key role in the regulation of the neuronal, and especially synaptic, microenvironment and are key elements in the brain parenchyma defense against oxidative and toxic insults (Sofroniew and Vinters, 2010; Heller and Rusakov, 2015). The number of visible astrocytes and the complexity of their processes may also be related to the extent of neural injury. Vanadium-induced astrogliosis has previously been reported in the cerebellum and hippocampus of adult rats exposed to sodium metavanadate for 5 days (Garcia et al., 2005) indicating a rapid response of astrocytes to this challenge. Astrocyte activation was also observed in the hippocampus and corpus callosum after vanadium exposure in rats of 2 weeks of age (Olopade and Connor, 2011; Todorich et al., 2011) and 3-week-old mice (Mustapha et al., 2014), indicating that reactive astrogliosis is a component of vanadium neurotoxicity. In the present study astrocytic response was observed at the onset of the exposure increasing till 12 months but with increasing vanadium exposure into old age, this response decreased. This could be as a result of repair process carried out by the astrocytes to enhance axonal regeneration and improve functional recovery after CNS injury (Kokaia et al., 1999; Simard and Nedergaard, 2004; Erschbamer et al., 2007). The fact that microglial activation remained pronounced into old age but less so for astrocyte may indicate that some intrinsic inflammatory pathway might have been selectively switched on in microglia after chronic vanadium exposure. Lu et al. (2014) showed that while proinflammatory cytokine AP-1 is involved in LPS-induced IL-1β expression and released by microglia and astrocytes. Resveratrol inhibits LPS-induced AP-1 activation in microglia but not astrocytes.

We had earlier reported that chronic exposure to vanadium over a life time led to memory loss (Folarin et al., 2016) after 3 months. In the present study, we detected decrease in neuronal number and apical dendrites of the hippocampal CA1 pyramidal cells which indicates cell death and severe decline in the number and availability of axonal inputs to dendritic ends. Temporary inactivation or lesions of the dorsal hippocampus have been reported to cause impairments in the acquisition and retrieval of spatial memory (Moser and Moser, 1998; Riedel et al., 1999). Thus we propose that the profound neuronal loss and hippocampal alteration observed will possibly result to memory impairment. Previous studies (Avila-Costa et al., 2004, 2006) have reported dendritic spine loss with glaring memory alteration after vanadium inhalation. Quantitative analysis of the cell count revealed significant reduction in neuronal number both in the hippocampal regions (CA1 and CA3) and the cerebellar cortex which support our previous findings (Mustapha et al., 2014; Azeez et al., 2016; Folarin et al., 2016). This result also shows more vulnerability of CA1 region to insults than CA3; this is because high intrinsic superoxide and endogenous ROS production occur in CA1 than CA3 region (Wilde et al., 1997; Wang et al., 2005). It is also reported that mitochondrial permeability transition pore of CA1 region is more sensitive to calcium homeostasis and this leads to active production of ROS (Mattiasson et al., 2003). Similar observations on relative CA1 vulnerability were reported following exposure of the hippocampus to alcohol (Tran and Kelly, 2003; Miki et al., 2004).

Chronic vanadium exposure as shown in this study produced cytotoxicity and morphological alterations of the pyramidal cells of the prefrontal cortex characterized by cell clustering, loss of layering pattern and cytoplasmic vacuolation suggesting deterioration of cell functioning which ultimately leads to destruction of cellular structures and cell death (Henics and Wheatley, 1999). The immuno-fluorescent staining of the cortical pyramidal cells with a nuclear DNA cell marker revealed cells with intact but shrunken nucleus and cytoplasmic loss. This result supports previous observations which suggest that vanadium cytotoxicity appears more pronounced than its apoptotic effects (Mustapha et al., 2014). We also noticed in the neurons of prefrontal cortex and CA1 region that vanadium neuropathologies of dendritic spine loss, cytotoxicity after 6–9 months resembled those of geriatric control brains at 15 and 18 months. This is indicative of neuronal degeneration occurring after vanadium exposure and consistent with previous studies (Calderón-Garcidueñas et al., 2012) which suggested that chronic exposure to environmental pollutants resulted in detection of old age associated lesions in children or young adult brain and thus connotes premature aging.

This study has shown a relative reversal of vanadium neurotoxicity both in the neuronal and glial cellular alteration and severity of activation after withdrawal from exposure to the metal. This could be as a result of reducing metal oxidative assault or repair processes which is compensating for possible early CNS damage (Woźniak et al., 2004). The data from the LA–ICP–MS also shows marked and progressive elimination of vanadium metal from the brain with increasing duration of withdrawal from treatment. It is noteworthy that we observed almost a complete reversal of memory loss profile in mice after 6–9 months of vanadium withdrawal (Folarin et al., 2016), however the present study shows that vanadium metal load in the brain and CNS lesion induced by vanadium neurotoxicity were not completely eliminated even though the functions may be almost fully restored back to normal. This may be as a result of surviving neurons functioning to compensate for the lost cells.

## Conclusion

We have shown that chronic administration of vanadium over a life time in mice resulted in metal accumulation which showed regional variabilities with time. With increase in the chronicity of the exposure, microglia activation rather than astrocytic activation becomes more predominant. The vanadium metal uptake and pathological effects were not completely eliminated from the brain even after a long time withdrawal from vanadium metal.

## Author Contributions

JOO and FO conceptualized this work from inception. JOO and JRC supervised ORF on aspects of this work. ORF did the mice experiments, feeding, treatment with vanadium and animal sacrifice, and slide preparation. AMS and ORF did the immunohistochemistry and double labeling while ORF and FO were involved in the processing of brain samples, histology and related data analysis. DGP, ORF and JRC were involved in the LA–ICP–MS analysis. All authors contributed to manuscript writing, analysis and correction.

## Conflict of Interest Statement

The authors declare that the research was conducted in the absence of any commercial or financial relationships that could be construed as a potential conflict of interest.

## References

[B1] Amorim F. A. C. Welz B. Costa A. C. S. Lepri F. G. Vale M. G. R. Ferreira S. L. C. (2007). Determination of vanadium in petroleum and petroleum products using atomic spectrometric techniques. Talanta 72, 349–359. 10.1016/j.talanta.2006.12.015 19071624

[B300] Avila-Costa M. R. Colín-Barenque L. Zepeda-Rodríguez A. Antuna S. B. Saldivaro L. Espejel-Maya G.. (2005). Ependymal epithelium disruption after vanadium pentoxide inhalation: a mice experimental model. Neurosci. Lett. 381, 21–25. 10.1016/j.neulet.2005.01.072 15882783

[B2] Avila-Costa M. R. Fortou T. I. Niñno-Cabrera G. Coliín-Barenque L. Bizarro-Nevares P. Gutieérrez-Valdez A. L.. (2006). Hippocampal cell alterations induced by the inhalation of vanadium pentoxide (V_2_O_5_) promote memory deterioration. Neurotoxicology 27, 1007–1012. 10.1016/j.neuro.2006.04.001 16684564

[B3] Avila-Costa M. R. Montiel-Flores E. Colin-Barenque L. Ordonñez J. L. Gutiérrez A. L. Niño-Cabrera H. G.. (2004). Nigrostriatal modifications after vanadium inhalation: an immunocytochemical and cytological approach. Neurochem. Res. 29, 1365–1369. 10.1023/b:nere.0000026398.86113.7d 15202766

[B4] Azeez I. A. Olopade F. Laperchia C. Andrioli A. Scambi I. Onwuka S. K.. (2016). Regional myelin and axon damage and neuroinflammation in the adult mouse brain after long-term postnatal vanadium exposure. J. Neuropathol. Exp. Neurol. 75, 843–854. 10.1093/jnen/nlw058 27390101

[B5] Block M. L. Calderón-Garcidueñas L. (2009). Air pollution: mechanisms of neuroinflammation and CNS disease. Trends Neurosci. 32, 506–516. 10.1016/j.tins.2009.05.009 19716187 PMC2743793

[B6] Block M. L. Hong J. S. (2007). Chronic microglial activation and progressive dopaminergic neurotoxicity. Biochem. Soc. Trans. 35, 1127–1132. 10.1042/bst0351127 17956294

[B8] Calderón-Garcidueñas L. Kavanaugh M. Block M. D’Angiulli A. Delgado-Chávez R. Torres-Jardón R.. (2012). Neuroinflammation, hyperphosphorylated tau, diffuse amyloid plaques, and down-regulation of the cellular prion protein in air pollution exposed children and young adults. J. Alzheimers Dis. 28, 93–107. 10.3233/JAD-2011-110722 21955814

[B10] Chen F. Ding M. Castranova V. Shi X. (2001). Carcinogenic metals and NF-κB activation. Mol. Cell. Biochem. 222, 159–171. 10.1007/978-1-4615-0793-2_19 11678598

[B14] Domingo J. L. (1996). Vanadium: a review of the reproductive and developmental toxicity. Reprod. Toxicol. 10, 175–182. 10.1016/0890-6238(96)00019-6 8738553

[B15] Erschbamer M. Pernold K. Olson L. (2007). Inhibiting epidermal growth factor receptor improves structural, locomotor, sensory, and bladder recovery from experimental spinal cord injury. J. Neurosci. 27, 6428–6435. 10.1523/jneurosci.1037-07.2007 17567803 PMC6672443

[B16] Evangelou A. M. (2002). Vanadium in cancer treatment. Crit. Rev. Oncol. Hematol. 42, 249–265. 10.1016/s1040-8428(01)00221-9 12050018

[B17] Folarin O. Olopade F. Onwuka S. Olopade J. (2016). Memory deficit recovery after chronic vanadium exposure in mice. Oxid. Med. Cell. Longev. 2016:4860582. 10.1155/2016/4860582 26962395 PMC4745327

[B18] Fortoul T. I. Rodriguez-Lara V. González-Villalva A. Rojas-Lemus M. Cano-Gutiérrez G. Ustarroz-Cano M. (2014). Inhalation of vanadium pentoxide and its toxic effects in a mouse model. Inorg. Chim. Acta 420, 8–15. 10.1016/j.ica.2014.03.027

[B19] Garcia G. B. Biancardi M. E. Quiroga A. D. (2005). Vanadium (V)-induced neurotoxicity in the rat central nervous system: a histo-immunohistochemical study. Drug Chem. Toxicol. 28, 329–344. 10.1081/dct-200064496 16051558

[B20] García G. B. Quiroga A. D. Stürtz N. Martinez A. L. Biancardi M. E. (2004). Morphological alterations of central nervous system (CNS) myelin in vanadium (V)-exposed adult rats. Drug Chem. Toxicol. 27, 281–293. 10.1081/dct-120037747 15478949

[B22] Haider S. S. Abdel-Gayoum A. A. el-Fakhri M. Ghwarsha K. M. (1998). Effect of selenium on vanadium toxicity in different regions of rat brain. Hum. Exp. Toxicol. 17, 23–28. 10.1191/096032798678907784 9491334

[B24] Hare D. J. Lee J. K. Beavis A. D. van Gramberg A. George J. Adlard P. A.. (2012). Three-dimensional atlas of iron, copper and zinc in the mouse cerebrum and brainstem. Anal. Chem. 84, 3990–3997. 10.1021/ac300374x 22462591

[B23] Hare D. Reedy B. Grimm R. Wilkins S. Volitakis I. George J. (2009). Quantitative elemental bio-imaging of Mn, Fe, Cu and Zn in 6-hydroxydopamine Parkinsonism mouse models. Metallomics 1, 53–58. 10.1039/b816188g

[B25] Heller J. P. Rusakov D. A. (2015). Morphological plasticity of astroglia: understanding synaptic microenvironment. Glia 63, 2133–2151. 10.1002/glia.22821 25782611 PMC4737250

[B26] Henics T. Wheatley D. N. (1999). Cytoplasmic vacuolation, adaptation and cell death: a view on new perspectives and features. Biol. Cell 91, 485–498. 10.1016/s0248-4900(00)88205-2 10572624

[B28] Kokaia Z. Airaksinen M. S. Nanobashvili A. Larsson E. Kujamäki E. Lindvall O.. (1999). GDNF family ligands and receptors are differentially regulated after brain insults in the rat. Eur. J. Neurosci. 11, 1202–1216. 10.1046/j.1460-9568.1999.00513.x 10103116

[B29] Kreutzberg G. W. (1996). Microglia: a sensor for pathological events in the CNS. Trends Neurosci. 19, 312–318. 10.1016/0166-2236(96)10049-7 8843599

[B30] Li H. Zhou D. Zhang Q. Feng C. Zheng W. He K.. (2013). Vanadium exposure-induced neurobehavioral alterations among Chinese workers. Neurotoxicology 36, 49–54. 10.1016/j.neuro.2013.02.008 23500660 PMC4160152

[B7] Lu C.-L. Tang S. Meng Z.-J. He Y.-Y. Song L.-Y. Liu Y.-P.. (2014). Taurine improves the spatial learning and memory ability impaired by sub-chronic manganese exposure. J. Biomed. Sci. 21:51. 10.1186/1423-0127-21-51 24885898 PMC4045917

[B31] Mattiasson G. Friberg H. Hansson M. Elmer E. Wieloch T. (2003). Flow cytometric analysis of mitochondria from CA1 and CA3 region of rat hippocampus reveals differences in permeability transition pore activation. J. Neurochem. 87, 532–544. 10.1046/j.1471-4159.2003.02026.x 14511130

[B32] Miki T. Harris S. J. Wilce P. A. Takeuchi Y. Bedi K. S. (2004). Effects of age and alcohol exposure during early life on pyramidal cell numbers in the CA1-CA3 region of the rat hippocampus. Hippocampus 14, 124–134. 10.1002/hipo.10155 15058490

[B33] Moser M. B. Moser E. I. (1998). Distributed encoding and retrieval of spatial memory in the hippocampus. J. Neurosci. 18, 7535–7542. 9736671 10.1523/JNEUROSCI.18-18-07535.1998 PMC6793256

[B34] Moskalyk R. R. Alfantazi A. M. (2003). Processing of vanadium: a review. Miner. Eng. 16, 793–805. 10.1016/s0892-6875(03)00213-9

[B35] Mustapha O. Oke B. Offen N. Sirén A. L. Olopade J. (2014). Neurobehavioral and cytotoxic effects of vanadium during oligodendrocyte maturation: a protective role for erythropoietin. Environ. Toxicol. Pharmacol. 38, 98–111. 10.1016/j.etap.2014.05.001 24927405

[B36] Ngwa H. A. Kanthasamy A. Jin H. Anantharam V. Kanthasamy A. G. (2014). Vanadium exposure induces olfactory dysfunction in an animal model of metal neurotoxicity. Neurotoxicology 43, 73–81. 10.1016/j.neuro.2013.12.004 24362016 PMC4062607

[B38] Olopade J. O. Connor J. R. (2011). Vanadium and neurotoxicity: a review. Curr. Top. Toxicol. 7, 33–39.

[B39] Olopade J. O. Fatola I. O. Olopade F. E. (2011). Vertical administration of vanadium through lactation induces behavioural and neuromorphological changes: protective role of vitamin E. Niger J. Physiol. Sci. 26, 55–60. 22314988

[B40] Pyrzyńska K. Wierzbicki T. (2004). Determination of vanadium species in environmental samples. Talanta 64, 823–829. 10.1016/j.talanta.2004.05.007 18969676

[B41] Ray R. S. Rana B. Swami B. Venu V. Chatterjee M. (2006). Vanadium mediated apoptosis and cell cycle arrest in MCF7 cell line. Chem. Biol. Interact. 163, 239–247. 10.1016/j.cbi.2006.08.006 16970931

[B42] Riedel G. Micheau J. Lam A. G. Roloff E. Martin S. J. Bridge H.. (1999). Reversible neural inactivation reveals hippocampal participation in several memory processes. Nat. Neurosci. 2, 898–905. 10.1038/13202 10491611

[B43] Sasi M. M. Haider S. S. el-Fakhri M. Gwarsha K. M. (1994). Microchromatographic analysis of lipid protein and occurrence of lipid peroxidation in various brain areas of vanadium exposed rats: a possible mechanism of vanadium neurotoxicity. Neurotoxicity 15, 413–420. 7991230

[B45] Saxena P. N. Arya J. Saxena N. Shukla A. (2013). Vanadium intoxication in albino rat based on haematobiochemistry and behaviouristic changes. Int. J. Environ. En. Manag. 4, 293–300.

[B46] Shrivastava S. Jadon A. Shukla S. (2007). Effect of tiron and its combination with nutritional supplements against vanadium intoxication in female albino rats. J. Toxicol. Sci. 32, 185–192. 10.2131/jts.32.185 17538242

[B48] Simard M. Nedergaard M. (2004). The neurobiology of glia in the context of water and ion homeostasis. Neuroscience 129, 877–896. 10.1016/j.neuroscience.2004.09.053 15561405

[B49] Soazo M. Garcia G. B. (2007). Vanadium exposure through lactation produces behavioural alterations and cns myelin deficit in neonatal rats. Neurotoxicol. Teratol. 29, 503–510. 10.1016/j.ntt.2007.03.001 17493788

[B50] Sofroniew M. V. Vinters H. V. (2010). Astrocytes: biology and pathology. Acta Neuropathol. 119, 7–35. 10.1007/s00401-009-0619-8 20012068 PMC2799634

[B51] Todorich B. Olopade J. O. Surguladze N. Zhang X. Neely E. Connor J. R. (2011). The mechanism of vanadium-mediated developmental hypomyelination is related to destruction of oligodendrocyte progenitors through a relationship with ferritin and iron. Neurotox. Res. 19, 361–373. 10.1007/s12640-010-9167-1 20237879

[B52] Tran T. D. Kelly S. J. (2003). Critical periods for ethanol-induced cell loss in the hippocampal formation. Neurotoxicol. Teratol. 25, 519–528. 10.1016/s0892-0362(03)00074-6 12972065

[B53] Tsuda M. Mizokoshi A. Shigemoto-Mogami Y. Koizumi S. Inoue K. (2004). Activation of p38 mitogen-activated protein kinase in spinal hyperactive microglia contributes to pain hypersensitivity following peripheral nerve injury. Glia 45, 89–95. 10.1002/glia.10308 14648549

[B54] Usende I. Leitner D. F. Neely E. B. Connor J. R. Olopade J. (2016). The deterioration seen in myelin related morphophysiology in vanadium exposed rats is partially protected by concurrent iron deficiency. Niger J. Physiol. Sci. 31, 11–22. 27574759

[B55] Wang X. Palb R. Chen X.-C. Limpeanchobb N. Kumara K. N. Michaelis E. K. (2005). High intrinsic oxidative stress may underlie selective vulnerability of the hippocampal CA1 region. Mol. Brain Res. 140, 120–126. 10.1016/j.molbrainres.2005.07.018 16137784

[B56] Wilde J. Pringle A. K. Wright P. Iannotti F. (1997). Differential vulnerability of the CA1 and CA3 subfields of the hippocampus to superoxide and hydroxyl radicals *in vitro*. J. Neurochem. 69, 883–886. 10.1046/j.1471-4159.1997.69020883.x 9231752

[B57] Woźniak A. Drewa G. Woźniak B. Schachtschabel D. O. (2004). Activity of antioxidant enzymes and concentration of lipid peroxidation products in selected tissues of mice of different ages, both healthy and melanoma-bearing. Z. Gerontol. Geriatr. 37, 184–189. 10.1007/s00391-004-0229-y 15224238

[B58] Zhen X. Torres C. Cai G. Friedman E. (2002). Inhibition of protein tyrosine/mitogen-activated protein kinase phosphatase activity is associated with d2 dopamine receptor supersensitivity in a rat model of Parkinson’s disease. Mol. Pharmacol. 62, 1356–1363. 10.1124/mol.62.6.1356 12435803

